# A retrospective study of Human Immunodeficiency Virus transmission, mortality and loss to follow-up among infants in the first 18 months of life in a prevention of mother-to-child transmission programme in an urban hospital in KwaZulu-Natal, South Africa

**DOI:** 10.1186/1471-2431-12-146

**Published:** 2012-09-10

**Authors:** Terusha Chetty, Stephen Knight, Janet Giddy, Tamaryn L Crankshaw, Lisa M Butler, Marie-Louise Newell

**Affiliations:** 1 Africa Centre for Health and Population Studies, University of KwaZulu-Natal, Mtubatuba, South Africa; 2 Department of Public Health Medicine, University of KwaZulu-Natal, Durban, South Africa; 3 McCord Hospital, Durban, South Africa; 4 Department of Epidemiology and Biostatistics, University of California, San Francisco, CA, USA; 5 Global Health Sciences, University of California, San Francisco, USA

**Keywords:** HIV-exposed infants, LTFU, Prevention-of-Mother-to-Child Transmission, Postnatal clinic

## Abstract

**Background:**

Follow up of Human Immunodeficiency Virus (HIV)-exposed infants is an important component of Prevention of Mother-to-Child Transmission (PMTCT) programmes in order to ascertain infant outcomes post delivery. We determined HIV transmission, mortality and loss to follow-up (LTFU) of HIV-exposed infants attending a postnatal clinic in an urban hospital in Durban, South Africa.

**Methods:**

We conducted a retrospective cohort study of infants born to women in the PMTCT programme at McCord Hospital, where mothers paid a fee for service. Data were abstracted from patient records for live-born infants delivered between 1 May 2008 and 31 May 2009. The infants’ LTFU status and age was based on the date of the last visit. HIV transmission was calculated as a proportion of infants followed and tested at six weeks. Mortality rates were analyzed using Kaplan-Meier (K-M), with censoring on 15 January 2010, LTFU or death.

**Results:**

Of 260 infants, 155 (59.6%) remained in care at McCord beyond 28 weeks: one died at < 28 days, three died between one to six months; 34 were LTFU within seven days, 60 were LTFU by six months. K-M mortality rate: 1.7% at six months (95% confidence interval (CI): 0.6% to 4.3%). Of 220 (83%) infants tested for HIV at six weeks, six (2.7%, 95% CI: 1.1% to 5.8%) were HIV-infected. In Cox regression analysis, late antenatal attendance (≥ 28 weeks gestation) relative to attending in the first trimester was a predictor for infant LTFU (adjusted hazards ratio = 2.3; 95% CI: 1.0 to 5.1; p = 0.044).

**Conclusion:**

This urban PMTCT programme achieved low transmission rates at six weeks, but LTFU in the first six months limited our ability to examine HIV transmission up to 18 months and determinants of mortality. The LTFU of infants born to women who attended antenatal care at 28 weeks gestation or later emphasizes the need to identify late antenatal attendees for follow up care to educate and support them regarding the importance of follow up care for themselves and their infants.

## Background

Globally, approximately 370 000 children were newly infected with Human Immunodeficiency Virus (HIV) in 2009; the vast majority in sub-Saharan Africa [1], mostly due to mother-to-child transmission. In resource-constrained settings, with little or no antiretroviral treatment, approximately one-third of HIV-infected children die before one year and more than half die before two years of age [2]. In South Africa (SA), where the 2008 antenatal HIV seroprevalence was 29% [3], the child mortality rate increased from 56 per 1000 live births in 1990 to 67 per 1000 in 2008 [4]; HIV was the main contributor to the rising child mortality, responsible for approximately 40% of under-five mortality in 2000 [5]. Between 2001 and 2006, in a rural area of northern KwaZulu-Natal, improving maternal access to antiretroviral therapy (ART) and Prevention of Mother-to-Child Transmission (PMTCT) interventions resulted in a 57% decline in infant mortality [6].

While PMTCT interventions in SA prior to 2010 focused only on the perinatal period [7], the updated guidelines now recommend continuing ART during the postnatal period while breastfeeding and the use of oral nevirapine for all infants born to HIV-infected women who are not on ART for life and still breastfeeding (until one week after cessation of breastfeeding) [8]. With the successful implementation of the current South African antenatal PMTCT programmes aiming to reduce vertical transmission of HIV to less than five percent [9], poor follow-up of HIV-exposed infants remains a major weakness, undermining the ability to support safe infant feeding practices and to measure infant HIV-free survival at 18 months. Hence, understanding the determinants of retention is important. In a public-funded urban hospital in Johannesburg, South Africa, almost half of infants born to HIV-infected mothers in a routine PMTCT service were lost to follow-up (LTFU) by two weeks of age [10]. At the time this study was conducted, the national guidelines in South Africa did not include routine HIV testing for infants at six weeks and recommended testing for infants at 12 months of age [10]. Similarly, studies from Zimbabwe, Uganda, and Malawi reported LTFU of infants born to HIV-infected mothers in PMTCT programmes of 41% at 12 months, 30% at 18 months, and 30% at 24 months, respectively [11-13]. In a hospital in Malawi 88% of infants who received nevirapine at the hospital were seen at the postnatal clinic [14]. More than half of these infants (n = 122, 54%) returned for their six month follow-up visit [14]. Early recognition of HIV-exposed infants is crucial given the higher incidence of infectious disease morbidity and mortality observed in HIV-infected and uninfected infants [2,15-18]. Further, early identification of HIV-infected infants is essential for initiation of ART, which can reduce early infant mortality by 76% and HIV disease progression by 75% [19].

In response to an increasing concern about LTFU of infants born to HIV-infected women in the PMTCT programme following delivery, in May 2008, McCord Hospital implemented a postnatal clinic for all HIV-infected mothers (with their infants) who had attended the PMTCT programme at the hospital. This new postnatal service provided an opportunity to characterize the frequency and correlates of LTFU of infants born to women attending the PMTCT programme at McCord Hospital.

## Methods

### Design and setting

We conducted a retrospective cohort study of infants born to HIV-infected women attending McCord Hospital, a state-subsidized hospital in Durban, which serves a population from a wide geographic catchment area. The patient population attending the hospital is predominantly a lower-middle-income, medically uninsured population. All postnatal services for HIV-exposed infants are free but the mothers receiving care paid a user fee of ZAR 160 (~$US24) per visit at the time.

The study population consisted of infants born to HIV-infected women who received antiretroviral prophylaxis or ART from the McCord Hospital PMTCT programme, and whose infants were born at the hospital or elsewhere between 1 May 2008 and 31 May 2009, and/or whose infants were presented for care following delivery to McCord Hospital.

### PMTCT care at McCord Hospital

In February 2008, the South African Department of Health (DoH) adopted a new PMTCT protocol, which was more aligned to the World Health Organization 2006 recommendations [7,20]. The main change to the national protocol was the addition of short course zidovudine from 28 weeks gestation, to the single dose (sd)-nevirapine at delivery for all pregnant women with CD4^+^ cell counts above 200 cells/mm^3^.

The PMTCT guidelines used at McCord Hospital, before the updated DoH guidelines were based on evidence from international studies [21,22]. Pregnant women with CD4^+^ cell counts above 200 cells/mm^3^ were initiated on prophylactic antiretroviral regimens, chosen according to the maternal viral load and the gestational age at first visit [21,22]. HIV-infected pregnant women were offered an elective caesarean section if their viral load was above 1000 copies/ml at 36 weeks (Giddy, J. Personal communication, June 2008). For women who did not attend antenatal services prior to delivery and were not receiving antiretroviral prophylaxis or therapy, the 2008 McCord Hospital guidelines were as follows: intrapartum sd-nevirapine with a postpartum seven day tail of zidovudine and lamivudine; in 2009 the guidelines were amended to include three-hourly intrapartum zidovudine. Infants received sd-nevirapine within 72 hours of delivery. Newborn infants were given four weeks zidovudine postpartum for the following reasons: (i) if the mother received no ART during pregnancy; (ii) if the maternal viral load was above 1000 copies/ml at 36 weeks gestation; or (iii) if the mother had received less than one month of ART prior to delivery. Those infants whose mothers received more than four weeks of ART antenatally were given zidovudine for one week post partum. All women received antenatal counselling regarding infant feeding options, and were supported in their choice.

In April 2010, the National PMTCT guidelines were revised to include triple ART for life for women with a CD4^+^ cell count of 350 cells/mm^3^ or less; or zidovudine from 14 weeks of pregnancy, intrapartum (sd)-nevirapine and three-hourly zidovudine, and a postpartum tail of sd-tenofovir and emtracitabine for women with a CD4^+^ cell count more than 350 cells/mm^3^[8]. The 2010 guideline on infant antiretroviral prophylaxis included six weeks of infant nevirapine for the non-breastfed infant or if the breastfeeding mother was receiving lifelong ART [8]. In breastfed infants, oral nevirapine was provided for the duration of breastfeeding until one week after cessation of breastfeeding [8].

### Patient visits

At McCord Hospital, HIV-infected pregnant women have a baseline CD4^+^ cell count and viral load investigations at their initial presentation for antenatal care. At each visit, women receive a comprehensive consultation that includes tuberculosis screening. The postnatal clinic provides the following care up to 18 months post delivery: maternal HIV care and ART provision, infant HIV testing at six weeks by polymerase chain reaction (PCR) and 18 month HIV antibody testing, primary health care for the mother and infant, which includes immunization, development and growth monitoring, reproductive health care, psychosocial support, and appropriate health promotion. Socio-demographic, biologic, clinical and immunologic data on mothers and their infants are recorded on standard clinic forms.

Infant HIV infection status is determined by PCR on dried blood spots (DBS Sample Collection Kit for Infant HIV PCR Tests – CCMT Programme, Lasec, SA) collected from heel prick at six weeks of age. A subsequent HIV PCR is collected at 14 weeks of age. For breastfed infants, an additional PCR assay is performed six weeks after cessation of breastfeeding. Infants who test negative for HIV at 14 weeks of age at the postnatal clinic are offered a rapid HIV test at 18 months using a Determine HIV 1/2 Test Abbott Laboratories, Abbott Park, IL and Sensa Tri-Line HIV 1/2/0 (Hitech Healthcare LTD, China). An infant was presumed HIV-1 uninfected if they had negative DNA PCR assays at six and 14 weeks of age. A child was classified as HIV-1 uninfected if both antibody tests were negative at or after 18 months.

For all patients who do not return for follow-up visit, three different attempts are made to contact the patient by phone. Reasons for not returning for care are documented when provided.

### Data collection

Routine data on antenatal history, care, treatment and delivery were collected from maternal antenatal and PMTCT records. Data were abstracted to a specific study data collection form and then entered into a database by a trained data capturer.

### LTFU outcome

Data on the infants’ follow-up care were obtained from their records. The infants’ LTFU status and age was assigned based on the date of their last clinic visit. Infants who missed a scheduled visit but subsequently presented for a consultation (either scheduled or unscheduled) to McCord Hospital were not considered lost to follow-up. Initial follow-up periods occurred at one, six, ten, and 14 weeks; but later changed to six, nine, 12, 15 and 18 months to coincide with the South African immunization schedule. A LTFU status was assigned if the infant did not return within two weeks of scheduled visits in the first five months or within one month of the later follow-up visits. Infants who were referred to other clinics or discharged or transferred from the clinic at 18 months were not considered LTFU.

### Mortality

The neonatal mortality rate is defined as the number of deaths in the first 28 completed days of life per 1000 live births in a given year or period and the perinatal mortality rate is defined as the number of stillbirths and deaths in the first week of life per 1000 live births [23].

### Statistical analysis

Observations were censored at 15 January 2010, LTFU, transfer or death, whichever occurred first. HIV transmission, child deaths and LTFU of infants were treated as time-to-event variables and were analyzed with Kaplan-Meier (K-M) methods [24]. Factors associated with LTFU of infants were recorded. Socio-demographic (age, race, employment and marital status), clinical (any illness during pregnancy, gestational age at first visit, obstetric sepsis, preterm labour, maternal mortality) immunological (CD4^+^ counts and viral loads) and biological (parity, mode of delivery, infant birth weight, sex of infant and feeding modality) factors were assessed to determine their association with LTFU of infants. Both single and multiple Cox Regression analyses were conducted to identify important predictors. Cox Proportional Hazards Regression analysis was used to retrieve baseline survival function and to model multivariable survival data. The Likelihood Ratio Test was used to assess the fit of the model. For multiple regressions, a backward elimination procedure used log-likelihood criteria with p > 0.1 for removing variables and p < 0.05 for entering variables. Second and third-born infants from multiple births (n = 7) were excluded from the analysis as it was assumed the factors associated with LTFU would be the same for infants of multiple births.

All analyses were performed in Stata/IC version 11 (Statacorp, College Station, Texas).

### Ethics

The study was approved by the University of KwaZulu-Natal Biomedical Research Ethics Committee (reference number BE160/08) and McCord Hospital Research Ethics Committee. Individual patient consent was not obtained for this retrospective study as patient contact was not necessary. Moreover, no patient identifiers were collected and data used for the study included only data collected for standard care at McCord Hospital.

## Results

In total, 272 HIV-infected mothers had deliveries between 1 May 2008 and 31 May 2009. Fourteen infants were excluded from the study due to missing records (n = 4), stillbirth deliveries (n = 4), duplicate entry (n = 1) and not meeting the inclusion criteria (n = 5). There were 258 deliveries resulting in 264 infants (251 singletons, five sets of twins and one set of triplets).

Median maternal age was 28.0 years (inter quartile range (IQR): 25.0 to 33.0 years). Almost two-thirds of mothers were employed and the majority were single. The baseline median maternal CD4^+^ cell count was 308 cells/mm^3^ (IQR: 192 to 420 cells/mm^3^). The median baseline viral load was 4320 copies/ml (IQR: 450 to 15 500 copies/ml). Twelve of 127 (9.4%) women with data had pulmonary tuberculosis and were receiving treatment (Table 1). There was only one maternal death during the study period (Table 1). The cause of the maternal death was reported as disseminated Kaposi’s Sarcoma (data not shown).

**Table 1:** Characteristics of pregnant HIV-infected women, 1 May 2008 to 31 May 2009, McCord Hospital

***Maternal characteristics***	N*	**n**	**%**	**95% Confidence Intervals**
**Race**	125			
Black (South African)		119	95.2	89.8 - 98.2
Asian		4	3.2	0.9 - 8.0
White		1	0.8	0.0 - 4.4
Black (other African)		1	0.8	0.0 - 4.4
**Parity**	247			
Primiparity		86	34.8	28.9 - 41.1
Multiparity		161	65.2	58.9 - 71.1
**Maternal CD4**^**+**^**count (cells/mm**^**3**^**)**	250			
< 200		68	27.2	21.8 - 33.2
200 – 499		147	58.8	52.4 - 65.0
≥ 500		35	14	9.9 - 18.9
**Maternal antiretroviral regimen**	258			
ART for life		128	49.6	43.4 - 55.9
PMTCT:				
Zidovudine, lamivudine. efavirenz from 28 weeks		85	32.9	27.2 - 39.0
Zidovudine from 28 weeks		40	15.5	11.3 - 20.5
sd-nevirapine + zidovudine and lamivudine for 7 days		4	1.6	0.4 - 3.9
Nevirapine only		1	0.4	0.0 - 2.1
**Booking gestational age, weeks**	246			
0-12		29	11.8	8.0 - 16.5
13-27		155	63.0	56.6 - 69.1
≥ 28		62	25.2	19.9 - 31.1
**Any illness during pregnancy**	247			
Yes		127	51.4	45.0 – 57.8
No		120	48.6	42.2 – 55.0
Pulmonary tuberculosis	127	12	9.4	5.0 -15.9
**Mode of delivery**	254			
Emergency C/S		74	29.1	23.6 - 35.1
Booked C/S		89	35.0	29.2 - 41.3
NVD		91	35.8	29.9 - 42.1
**Maternal mortality**	256			
No		255	99.6	97.8 – 100
Yes		1	0.4	0.0 - 2.2

N* The number of mothers across categories of maternal characteristics may not add up to 258 due to missing data.

Nearly half of the women (n = 128, 49.6%) included in the study were on ART for life (Table 1). Of the 106 women for whom the date of ART initiation was available, 97 (91.5%) were initiated on ART for life in the current pregnancy. The other 130 women (50.4%) were prescribed ARV prophylaxis for PMTCT; 85 (32.9%) were given interrupted triple therapy (zidovudine, lamivudine and efavirenz) from 28 weeks gestation until delivery, 40 (15.5%) were prescribed zidovudine from 28 weeks, and four (1.6%) received sd-nevirapine at delivery. Only one (0.4%) patient had sd-nevirapine alone due to late attendance in labour. All ARV prophylaxis for PMTCT was followed by a seven day tail of zidovudine and lamivudine. Most women (n = 155, 63.0%) attended their first antenatal visit at McCord Hospital between 13 to 27 weeks of gestation.

The median length of infant follow-up at McCord Hospital in this study was 9.1 months (IQR: 1.6 to 12.0 months) (Table 2). Of the 89 infants delivered through elective caesarean sections, 44.9% (n = 40) of caesarean sections were for women with viral loads above 1000 copies/ml at 36 weeks. The median birth weight was 3080 grams (IQR: 2780 to 3380 grams). There were 32 low birth weight infants (< 2500 grams) (12.8%; 95% CI: 8.9% to 17.6%). The median gestational age at birth was 38.0 weeks (IQR: 38.0 to 39.0 weeks). There were 189 infants born at term at or after 37 weeks (89.2%, 95% CI: 84.2% to 93.0%). Most infants (n = 223, 98.7%) received nevirapine and zidovudine as antiretroviral prophylaxis following delivery.

**Table 2:** Characteristics of HIV-exposed infants at McCord Hospital, 1 May 2008 to 31 May 2009

***Infant characteristics***	N*	**n**	**%**	**95% Confidence Interval**
**Observed length of follow-up**				
Median, months (IQR)		9.1 (1.6 - 12.0)		
**Sex**	**251**			
Male		122	48.6	42.3 - 55.0
Female		129	51.4	45.0 - 57.7
**ARV prophylaxis at birth**	**226**			
Nevirapine and zidovudine		223	98.7	96.2 - 99.7
**Gestational age at delivery, weeks**	**212**			
Mean (median)		38.2 (38.0)		37.8 – 38.2
< 37		23	10.8	7.0 - 15.8
≥ 37		189	89.2	84.2 - 93.0
**Birth weight (g)**	**250**			
Mean (median)		3052.3 (3080)		
< 2500		32	12.8	8.9 – 17.6
≥ 2500		218	87.2	82.4 – 91.1

N** the number of infants across each category of characteristics do not add up to 264 due to missing data.

### Proportion of infants by feeding category at birth

The majority of the 264 infants were reported to be exclusively formula-fed at birth (97.5%; 95% CI: 94.7% to 99.1%). The median duration of exclusive breastfeeding in the five breastfed infants was 18.5 weeks (IQR: 13.0 to 24.0 weeks). Exclusively breastfeeding was slightly longer than the median duration of exclusive formula feeding at 13.0 weeks (IQR: 6.0 to 14.0 weeks) (data not shown).

### HIV transmission

At 6 weeks 220 (83.0%) infants returned for HIV testing by PCR, and 6 (2.7%, 95% CI: 1.0% – 5.8%) were found to be HIV positive. None of the 13 children who were followed-up until 18 months of age were diagnosed as HIV-infected (95% CI: 0.0% to 3.6%) (data not shown).

### Mortality of infants from birth to five months

Among 264 infants in the cohort, four (1.5%) were known to have died. The crude mortality rate at six months was 15.2 per 1000 live births (95% CI: 4.1 to 38.3 per 1000 live births). There were eight perinatal deaths (four stillbirths). The perinatal mortality rate was 30.3 per 1000 live births (95% CI: 13.2 to 58.8 per 1000 live births). The neonatal mortality rate was 3.8 per 1000 live births (n = 1; 95% CI: 0.1 to 21.2 per 1000 live births). In Kaplan-Meier analyses, an estimated 1.7% of infants (95% CI: 0.6% to 4.3%) would have died by six months of age. The HIV-infection status and cause of death was unknown for three of the infants who died. One infant who died was diagnosed with meningitis at a tertiary level referral hospital. One infant who died was presumed HIV-uninfected. Two deceased infants were exclusively formula fed from birth; one infant was given formula and solids from two months. No feeding data was available for the remaining deceased infant (data not shown).

### LTFU

Of the remaining 260 live-born infants, 105 (40.4%; 95% CI: 34.4% to 46.6%) were LTFU, 155 infants (59.6%; 95% CI: 53.4% to 65.6%) remained in care at McCord Hospital beyond 28 weeks. Of these 155 infants, 13 infants (8.4%, 95% CI: 4.5% - 13.9%) were followed-up until 18 months of age. Three infants (1.9%, 95% CI: 0.4% - 5.6%) were transferred to other clinics. The period of greatest LTFU was in the first week of life (n = 34) with an incidence rate of 14.5 per 100 child-weeks (95% CI: 10.3 to 20.3 per 100 child-weeks). The LTFU rate declined steadily after the first week of life. From one to ten weeks, LTFU was 1.8 per 100 child-weeks (95% CI: 1.3 to 2.5 per 100 child-weeks). Sixteen infants were LTFU between 11 and 19 weeks (incidence rate 1.0; 95% CI: 0.6 to 1.6 per 100 child-weeks). The LTFU rate between 20 to 28 weeks was 0.6 per 100 child-weeks (95% CI: 0.3 to 1.2 per 100 child-weeks). The LTFU rate was reduced to 0.3 per 100 child-weeks (95% CI: 1.0 to 1.4 per 100 child-weeks) thereafter (data not shown).

### Maternal socio-demographic, clinical and immunologic factors associated with LTFU of infants

In multivariable analyses (excluding second and third infants of multiple births), the 31 infants born to mothers who had their first antenatal visit at or after 28 weeks were 2.3 times more likely to be LTFU, relative to infants whose mothers booked in their first trimester (95% CI: 1.0 to 5.1; p = 0.044) ( Additional file 1): Table 3. Maternal parity, age less than 30 years, being single and unemployed, and having a CD4^+^ cell count 500 cells/mm^3^ or more were not significantly associated with LTFU due to lack of statistical power. The reasons for LTFU of infants was missing in the majority of cases (n = 83; 79.8%).

## Discussion

In an urban South African setting, the integrated mother-child postnatal clinic at McCord Hospital achieved good outcomes in the first 13 months of its inception. These outcomes included infant HIV testing of 83% and HIV transmission risk of 2.7% at six weeks in returning HIV-exposed infants whose mothers received PMTCT at McCord Hospital. However, LTFU by six months was substantial, limiting our ability to determine HIV-free survival at 18 months.

In developed countries vertical transmission of HIV to infants occurs in 1-2% of pregnancies in HIV infected women, achieved through a combination of interventions, including antiretroviral therapy regimens that optimally suppress viral load, elective Caesarean section and complete avoidance of breastfeeding [25-30]. In developing countries the caesarean section proportion ranges from 3% to 12.6% [11,12,31]. The high proportion of caesarean sections at McCord Hospital may reflect the ability of the women in this setting to choose the best possible care for their infants and the hospital's capacity to deliver a comprehensive package of interventions to reduce the vertical transmission of HIV.

Although the rate of LTFU at McCord Hospital was comparable to other PMTCT programmes in public settings in sub-Saharan Africa [11,14,32-34], the high rate of early dropout in our setting may in part be due to women changing to service providers nearer to their residences. The period of greatest attrition was in the first week of life. This data may support the explanation of mothers returning to clinics in closer proximity to their homes. A similar finding was noted in a PMTCT programme at a centralized hospital in Malawi [14]. The authors suggested that the LTFU of mother-infant pairs may have been due to women from rural areas returning to their peripheral clinics following delivery [14]. The revised South African PMTCT guidelines may improve the follow-up of mothers and infants in this setting as HIV-infected women attend for their own health, and infants who are breastfed beyond six weeks require repeated access to nevirapine until one week after cessation of breastfeeding.

Late antenatal presentation (28 weeks of gestation or later) was a strong predictor of LTFU. There may be several reasons for late antenatal clinic attendance at McCord Hospital: women may have been minimizing expenses related to antenatal care; lack of awareness of the value of early antenatal care; or poor maternal health seeking behaviours. These results highlight the importance of identifying late antenatal attendees to determine the risk factors which contribute to attrition following delivery. Moreover, the fee-paying nature of the hospital needs to be taken into account when assessing the attrition of late antenatal attendees.

In prior studies, poor socio-demographic circumstances have been associated with patient attrition. In Malawi HIV-exposed infants born to parents who were less educated and in farming occupations were more likely to be LTFU [11]. In our multivariable model, there was no association between socio-demographic characteristics and infant LTFU (maternal employment and marital status). Since the parental level of education was not routinely collected at the time this study was conducted, the association with LTFU could not be assessed. In the Ugandan study lack of understanding of the importance of follow-up was noted as a reason for attrition [12]. In Johannesburg, maternal unemployment, geographical relocation and lack of paternal support were noted as reasons for poor retention [10]. Maternal education and support regarding the importance of follow-up care for their own and their infants’ health may improve patient retention and facilitate early diagnosis of infant morbidity and HIV transmission risk.

This study demonstrated that relative to infants of HIV-infected women with CD4 ^+^ counts 200 cells/mm^3^ or less, infants born to mothers with CD4^+^ counts above 200 cells/mm^3^ were more likely to be lost to follow-up. This finding was not statistically significant. Maternal well-being may be a risk factor for poor retention in our setting as mothers do not perceive themselves or their infants to be at risk for disease. These mothers may be less likely to seek health care. A similar correlation between infant wellbeing and loss to follow-up was shown in a study in rural Uganda, where infant illness was a protective against loss to follow-up in the PMTCT programme [12].

A key strength of this study was the ability to determine if patients were not returning for care. Clinic staff at McCord Hospital routinely contacted patients if they missed a scheduled appointment at the clinic, noting reasons for the missed appointment and rescheduling another. If these patients decided to attend other health care facilities, they were not considered LTFU as the reason for their non-attendance was known. Moreover, routine monitoring allowed early detection of infants requiring follow-up care.

A limitation of this study was the selection of the study population. According to the selection criteria, only infants whose mothers attended the PMTCT programme at McCord Hospital, and/or were delivered at McCord Hospital, and/or were brought back to the hospital for follow-up care were included in the study. Accordingly, the outcomes of infants who were not brought back to McCord Hospital for further care remained unknown. In addition, since the service was not available, the study could not include a comparison group of infants born to HIV-uninfected women from McCord Hospital. Inclusion of this comparison group would have made it possible to determine the background risk of HIV-exposed uninfected infants in terms of loss to follow-up. Finally, errors in the routine maternal and infant records and assignment of LTFU status may have constituted information bias.

The results of this study may not be generalizable to the HIV-exposed infant population in the public sector in South Africa. Moreover, formula feeding was reported as the predominant infant feeding modality. The impact of feeding practices on infant mortality requires further consideration. The results from this new integrated postnatal mother and baby clinic at McCord Hospital provide important lessons for other PMTCT services in developing settings. The attrition of HIV-exposed infants, particularly those born to women who attended antenatal services at McCord Hospital after 28 weeks highlights the need to identify late antenatal attendees for follow up care and to determine the HIV transmission risk and mortality of HIV-exposed infants.

## Conclusions

As efforts are made to improve rates of early HIV detection at six weeks of age and subsequent provision of care and treatment to HIV-exposed children in sub-Saharan Africa, it is essential that we develop better understanding of the determinants of retention and refine our strategies for retaining children who are at the greatest risk of morbidity and mortality in care. This preliminary study assessed a new postnatal service with its accompanying problems, further study is recommended to determine changes in the follow-up rates of infants.

## Abbreviations

ART: AntiRetroviral Therapy; CI: Confidence Interval; DBS: Dried Blood Spot; HIV: Human Immunodeficiency Virus; IQR: InterQuartile Range; K-M: Kaplan-Meier; LTFU: Loss To Follow-Up; PCR: Polymerase Chain Reaction; PMTCT: Prevention Of Mother-To-Child Transmission; SA: South Africa.

## Competing interests

The authors declare that they have no competing interests.

## Authors’ contributions

TC, principal investigator, contributed to the study conception and implementation, analyzed and interpreted the data, prepared the manuscript and supervised the project. JG, TLC and LMB contributed to the study conception, implementation, data interpretation and manuscript preparation. SK and MLN contributed to the study conception, data interpretation, and manuscript preparation. All authors read and approved the final manuscript.

## Authors’ information

TC FCPHM. SK FCPHM. JG MFamMed. TLC PhD. LMB PhD. MLN PhD.

## Funding

The Africa Centre for Health and Population Studies receives core funding from the Wellcome Trust.

## Pre-publication history

The pre-publication history for this paper can be accessed here:

http://www.biomedcentral.com/1471-2431/12/146/prepub

## Supplementary Material

Additional file 1 **Table 3.** Cox regression analysis of factors associated with LTFU of infants at McCord Hospital. Click here for file
