# Supplementary material for: A retrospective study of Human Immunodeficiency Virus transmission, mortality and loss to follow-up among infants in the first 18 months of life in a prevention of mother-to-child transmission programme in an urban hospital in KwaZulu-Natal, South Africa
Source: BMC Pediatr. 2012 Sep 10;12:146. doi: 10.1186/1471-2431-12-146 (PMC3468389; doi:10.1186/1471-2431-12-146)
Supplement: Additional file 1 — Table 3. Cox regression analysis of factors associated with LTFU of infants at McCord Hospital. [file 1471-2431-12-146-S1.docx]

**Table 3: Cox regression analysis of factors associated with LTFU of infants at McCord Hospital**

| ***Characteristics*** | **Live-born infants** | **Infant LTFU** | **%** | **Unadjusted**  **Hazards Ratio**  **(95% CI)** | **p-value** | **Adjusted**  **Hazards Ratio**  **(95% CI)** | **p-value** |
| --- | --- | --- | --- | --- | --- | --- | --- |
| **Maternal age** |  |  |  |  |  |  |  |
| ≥ 30 years | 107 | 36 | 33.6 | 1.0 |  | 1.0 |  |
| < 30 years | 144 | 68 | 47.2 | 1.5 | 0.061 | 1.3 | 0.280 |
|  |  |  |  | (1.0 – 2.2) |  | (0.8 – 2.1) |  |
| **Marital status** |  |  |  |  |  |  |  |
| Married | 37 | 12 | 32.4 | 1.0 |  | 1.0 |  |
| Single | 210 | 89 | 42.4 | 1.4 | 0.254 | 1.2 | 0.526 |
|  |  |  |  | (0.8 – 2.6) |  | (0.6 – 2.4) |  |
| **Race** |  |  |  |  |  |  |  |
| Unknown | 130 | 65 | 50.0 | 1.0 |  | 1.0 |  |
| Black (South African) | 117 | 37 | 31.6 | 0.6 | 0.007 | 0.6 | 0.035 |
|  |  |  |  | (0.4 – 0.9) |  | (0.4 – 1.0) |  |
| **Employed** |  |  |  |  |  |  |  |
| Yes | 162 | 65 | 40.1 | 1.0 |  | 1.0 |  |
| No | 84 | 34 | 40.5 | 1.0 | 0.948 | 0.9 | 0.636 |
|  |  |  |  | (0.7 – 1.5) |  | (0.6 – 1.4) |  |
| **Gestational age at booking** |  |  |  |  |  |  |  |
| 0 – 12 weeks | 29 | 8 | 27.6 | 1.0 |  | 1.0 |  |
| 13 - 27 weeks | 151 | 58 | 38.4 | 1.4 | 0.343 | 1.6 | 0.246 |
|  |  |  |  | (0.7 – 3.0) |  | (0.7 – 3.4) |  |
| ≥ 28 week | 61 | 31 | 50.8 | 2.0  (0.9 – 4.4) | 0.079 | 2.3  (1.1 – 5.1) | 0.044 |

| **Table 3 (contd.)** |  |  |  |  |  |  |  |
| --- | --- | --- | --- | --- | --- | --- | --- |
| ***Characteristics*** | **Live-born infants** | **Infants LTFU** | **%** | **Unadjusted**  **Hazards Ratio**  **(95% CI)** | **p-**  **value** | **Adjusted**  **Hazard Ratio**  **(95% CI)** | **p-**  **value** |
| **CD4^+^ count (cells/mm^3^)** |  |  |  |  |  |  |  |
| < 200 | 66 | 20 | 30.3 | 1.0 |  | 1.0 |  |
| 200 - 499 | 144 | 59 | 40.9 | 1.5 | 0.132 | 1.5 | 0.118 |
|  |  |  |  | (0.9 – 2.5) |  | (0.9 – 2.6) |  |
| ≥ 500 | 35 | 18 | 51.4 | 1.9 | 0.041 | 1.6 | 0.142 |
|  |  |  |  | (1.0 – 3.7) |  | (0.9 – 3.1) |  |
| **Parity** |  |  |  |  |  |  |  |
| Multiparity | 158 | 60 | 38.0 | 1.0 |  | 1.0 |  |
| Primiparity | 84 | 41 | 48.8 | 1.4 | 0.121 | 1.4 | 0.212 |
|  |  |  |  | (0.9 – 2.0) |  | (0.8 – 2.2) |  |

Number of subjects = 253

Number of observations = 253

Number of failures = 104

Time at risk = 61 163

Logistic regression chi square (19 df) = 54.78

Log likelihood = -517. 76801

Prob > chi square = 0.0000

The original models considered all the biological and socio-economic variables listed in table 1.

Log likelihood criteria p >0.1 for the following exposure variables; hence eliminated from the final model

Maternal death; smoking; illicit drug use; maternal baseline viral load (taken at the first antenatal visit); maternal illness during pregnancy, maternal antiretroviral regimen during pregnancy; preterm labour; obstetric sepsis; maternal mortality; feeding method at birth reported by mother; sex of infant; and infant birth weight
